# Relationship between the Magnitude of Intraocular Pressure during an Episode of Acute Elevation and Retinal Damage Four Weeks later in Rats

**DOI:** 10.1371/journal.pone.0070513

**Published:** 2013-07-29

**Authors:** Bang V. Bui, Abrez H. Batcha, Erica Fletcher, Vickie H. Y. Wong, Brad Fortune

**Affiliations:** 1 Department of Optometry and Vision Sciences, University of Melbourne, Melbourne, Australia; 2 National Vision Research Institute, Carlton, Victoria, Australia; 3 Department of Anatomy and Neuroscience, University of Melbourne, Melbourne, Australia; 4 Discoveries in Sight Research Laboratories, Devers Eye Institute and Legacy Research Institute, Legacy Health, Portland, Oregon, United States of America; University of Rochester, United States of America

## Abstract

**Purpose:**

To determine relationship between the magnitude of intraocular pressure (IOP) during a fixed-duration episode of acute elevation and the loss of retinal function and structure 4 weeks later in rats.

**Methods:**

Unilateral elevation of IOP (105 minutes) was achieved manometrically in adult Brown Norway rats (9 groups; n = 4 to 8 each, 10–100 mm Hg and sham control). Full-field ERGs were recorded simultaneously from treated and control eyes 4 weeks after IOP elevation. Scotopic ERG stimuli were white flashes (−6.04 to 2.72 log cd.s.m^−2^). Photopic ERGs were recorded (1.22 to 2.72 log cd.s.m^−2^) after 15 min of light adaptation (150 cd/m^2^). Relative amplitude (treated/control, %) of ERG components versus IOP was described with a cummulative normal function. Retinal ganglion cell (RGC) layer density was determined post mortem by histology.

**Results:**

All ERG components failed to recover completely normal amplitudes by 4 weeks after the insult if IOP was 70 mmHg or greater during the episode. There was no ERG recovery at all if IOP was 100 mmHg. Outer retinal (photoreceptor) function demonstrated the least sensitivity to prior acute IOP elevation. ERG components reflecting inner retinal function were correlated with post mortem RGC layer density.

**Conclusions:**

Retinal function recovers after IOP normalization, such that it requires a level of acute IOP elevation approximately 10 mmHg higher to cause a pattern of permanent dysfunction similar to that observed during the acute event. There is a ‘threshold’ for permanent retinal functional loss in the rat at an IOP between 60 and 70 mmHg if sustained for 105 minutes or more.

## Introduction

Elevated intraocular pressure (IOP) is an important risk factor for development of glaucomatous optic neuropathy yet the specific mechanisms and pathophysiological sequence by which IOP damages the optic nerve remain unknown. Thus, experimental models of glaucoma are necessary to determine the fundamental aspects of its pathophysiology. Naturally, nearly all such models are based on elevated IOP, which is typically induced in one eye of each animal or in some cases observed bilaterally in heritable forms of glaucoma [1]–[4]. Some tissue responses to elevated IOP occur relatively rapidly (e.g. mechanical effects and blood flow changes within seconds to minutes [5]–[7]; disruption of axonal transport within hours to days [8], [9] or genomic and proteomic changes within days to weeks) [10], [11], whereas others may take substantially longer to develop (such as remodeling of connective tissues like the lamina cribrosa, peripapillary sclera and glial septa within the optic nerve) [12], [13]. Thus varying the duration of IOP elevation can be informative insofar as it enables responses to relatively shorter duration IOP ‘spikes’ to be contrasted with those occurring after more chronic periods of IOP elevation. Indeed, it has been proposed that undetected IOP spikes can, over time, promote injury to the optic nerve and retina [14].

It is also important to discriminate between changes due to elevation of ambient IOP per se (i.e. during the episode) versus those that represent more permanent changes due either to a previous IOP spike or chronic elevation. One such example is the thickness of the optic nerve head (ONH) neural rim tissue, which is a common clinical means of structural assessment in glaucoma. Experimental studies in animals indicate that chronic IOP elevation leads to rim thinning as it does in human glaucoma [15]–[17]. However, it is also known that acute IOP elevation causes substantial rim thinning due directly to ONH conformational changes during episodes of elevated IOP [6], [18], [19]. Thus it is important to consider the degree to which the changes reported in chronic studies represent the ambient IOP at the time of testing versus more permanent changes reflecting chronic disease processes.

We have previously evaluated retinal functional changes during an acute episode (105 minutes) of IOP elevated to varying levels. [20] The results of that study demonstrated that *during* IOP elevation, changes to the ganglion cell component (scotopic threshold response, STR) of the dark adapted electroretinogram (ERG) were observable at moderate IOP levels (30–50 mm Hg) [20]. At higher levels of IOP elevation (80–100 mm Hg) ERG changes were more widespread, and involved ON-bipolar cell (b-wave) and photoreceptoral (a-waves) mediated features of the response waveform. Overall, the pattern of results demonstrated that there is a gradient of ERG attenuation, from inner to outer retina, with increasing level of acute IOP elevation. In the current study we evaluate retinal function using ERG, as well as retinal structure by post mortem histology, 4 weeks after the initial episode of acute IOP elevation. Thus we determine the degree of retinal function recoverable 4 weeks later (as compared with changes measured during the episode of IOP elevation) and the degree of permanent retinal damage (measured by both ERG and histology) as a function of IOP spike magnitude.

## Materials and Methods

The methodology employed in this study has been described in detail in our previous publication [20] and are summarized here in brief.

### Animals

All experiments conformed to the Association for Research in Vision and Ophthalmology Statement for the Use of Animals in Ophthalmic and Vision Research and were approved and monitored by the Institutional Animal Care and Use Committee at Legacy Health. Adult Brown-Norway rats between 10 and 12 weeks of age (180–260 g, Charles River Laboratories Inc., Willmington, MA, USA) were maintained in a 22°C, 12-h light (<40 lux)/12-h dark environment. Normal rat chow and water were available *ad libitum*.

### Acute IOP Elevation

Animals were dark adapted overnight (≥12 hrs) and prepared for cannulation and ERG recording under dim red light (wavelength >600 nm). Animals were anaesthetized with an intramuscular thigh injection of a mixture of ketamine (55 mg kg^−1^, Ketaset, Fort Dodge Animal Health, Fort Dodge, IA, USA), xylazine (5 mg kg^−1^, X-ject E, Phoenix Scientific Inc. St Joseph, MO, USA) and acepromazine maleate (1 mg kg^−1^, Aceproject, Phoenix Scientific Inc. St Joseph, MO, USA). Additional anesthesia was provided via the same route at 45 minute intervals (ketamine : xylazine : acepromazine 30∶2:1 mg/kg).

Mydriasis (≥ 4 mm) was induced with tropicamide (0.5%, Alcon Laboratories Inc., Fort worth, TX, USA) and phenylephrine (2.5%, Bausch and Lomb Pharmaceuticals Inc., Tampa, FL, USA). Corneal anesthesia was achieved with a drop of proparacaine hydrochloride (0.5%, Alcon Laboratories Inc.). Animals were lightly secured to a stage equipped with a water heat pad (TP500 T/Pump, Gaymar Industries, Orchard Park, NY, USA) to maintain body temperature between 37 and 38°C.

Following electrode placement (see below) the vitreous chamber (VC) was cannulated with a 30-gauge needle through the sclera superiorly, approximately 1 mm behind the limbus, at an angle of 45° to avoid contact with the lens. The cannula was connected via a pressure transducer (MX860, Medex Inc. Carlsbad, CA) to a reservoir filled with sterile balanced salt solution (BSS), the height of which was pre-calibrated for manometric control of IOP. Throughout the experiment IOP was monitored continuously and maintained within 1 mmHg of the target pressure (Propaq Encore model 206EL, Protocol Systems, Inc., Beaverton, OR). Immediately following the period of acute IOP elevation, eyes were assessed by direct ophthalmoscopy for complications including vitreous hemorrhage, retinal detachment or crystalline lens puncture (∼12% in total, which were then excluded from further analysis).

Ten groups of animals were used in this study and their final sample sizes were as follows: sham (n = 7), IOP = 10 mm Hg (n = 4), 20 (n = 5), 30 (n = 5), 40 (n = 5), 50 (n = 8), 60 (n = 7), 70 (n = 6), 80 (n = 6) and 100 mm Hg (n = 4). Target IOP level was maintained for a total of 105 minutes in every case and the ERG data collected during IOP elevation have been previously reported [20]. A broad-spectrum antibiotic ointment was applied to the treated eye (bacitracin and polymixin B sulfate, AK-POLY-BAC, Akorn Inc., Buffalo Grove, IL, USA) and animals recovered from anesthesia on a warm water pad (Gaymar Industries Inc., Orchard Park, NY, USA). ERGs were then recorded 4 weeks later using the same anesthesia regimen described above and the ERG recording protocol detailed in the next section. As previously reported [20], baseline IOP and arterial blood pressure in a subgroup of naïve rats was consistent with prior findings for adult Brown-Norway rats under ketamine anesthesia [21].

### Electroretinography

Four weeks after the episode of acute IOP elevation, full-field ERGs were recorded simultaneously from both experimental and fellow control eyes, as previously described, [20] using a UTAS-E3000 system (LKC Technologies, Gaithersburg, MD, USA). Stimuli were calibrated (Spectra Pritchard PR-1980B, Photo Research, Chatsworth, CA, USA) brief white flashes (xenon arc discharge, x = 0.32, y = 0.33) delivered via a Ganzfeld integrating sphere.

Scotopic threshold responses were obtained for flash intensities ranging from −6.04 to –5.36 log cd.s.m^−2^ in 0.2 log unit increments, by averaging 40–60 responses per intensity (60 for the dimmest and 40 for the higher intensities), with an interstimulus interval of 2 s. Scotopic ERGs (from −3.30 to 2.72 log cd.s.m^−2^) were recorded as single flash responses, with the interstimulus interval progressively lengthened from 10 to 120 s to allow complete recovery of the b-wave. Dark-adapted recordings were followed by 15 minutes adaptation to a white 150 cd.m^−2^ background. Photoptic responses (20 repeats, 2 s interstimulus interval) were recorded for intensities between 1.22 and 2.72 log cd.s.m^−2^ in 0.5 log unit increments.

### Tissue Preparation and Histopathology

At the end of experimentation animals were deeply anesthetized using the cocktail described above. Tissues were fixed by transcardial heparinization and perfusion with 125 ml of cold, 4% paraformaldehyde in 0.1 M phosphate buffered (pH 7.4) over 10 minutes. After enucleation and placement of incisions to mark orientation, all eyes were also post-fixed by immersion in the same 4% paraformaldehyde solution then processed for paraffin embedding. Longitudinal sections (6 µm thick) were cut through the globe along the anterior–posterior axis. Thus, retinal sections were vertically oriented, containing both inferior and superior retina. Sections were deparaffinized and rehydrated, stained with 0.1% hematoxylin and eosin (Sigma-Aldrich, St. Louis, MO) mounted for microscopy and photographed. Sections closest to the plane through the center of the anterior optic nerve were used to compare experimental with control eyes, thus matching lateral eccentricity as best as possible. Retinae were photographed using a Zeiss Axioplan Microscope (40 oil objective, Oberkochen, Ger) starting at the periphery near the ora serrata and progressing back to the optic nerve. Cells in the retinal ganglion cell (RGC) layer were counted manually by masked observer and then averaged to obtain a measurement of linear density (cells/mm). Ganglion cell density was further analysed by expressing the value in the experimental (IOP-elevated) eye as a percentage of its contralateral control eye. This was then compared to the 95% confidence limits, established for the sham group. Data falling outside of these confidence limits can be considered to be significant with an alpha-value of 0.05.

### Data Analysis

#### Scotopic threshold response, STR

The amplitude of the positive STR (pSTR) was measured from baseline to the initial peak and negative STR (nSTR) was measured from the baseline to the trough after the pSTR. Relative amplitude (treated/control, %) between –5.71 to –5.36 log cd.s.m^−2^ was evaluated for both the pSTR and nSTR.

#### Photoreceptor response, P3

The initial portion of the photoreceptoral a-wave was modeled using a delayed Gaussian function [22], [23]. This model returns a maximum response amplitude (Rm*_P3_*, µV), a gain (S, m^2^.cd^−1^s^−3^) and a time delay (t_d_, s) parameter via minimization of the root mean square error of the model fit to the set of 4 brightest flash responses for each eye using the Solver module of an Excel spreadsheet (Microsoft, Redmond, WA).

#### Rod bipolar cell response, scotopic P2

The scotopic P2 was isolated by subtracting the modeled P3 [24] followed by removing band-pass filtered (−3 dB at 50 and 280 Hz) oscillatory potentials (OPs). Peak amplitude was plotted against stimulus intensity and fitted with a hyperbolic function [25], [26], to return the maximum amplitude V*_max_* (µV), a sensitivity parameter *K* (log cd.s.m^−2^, intensity at half V*_max_*) and an exponent *n* (slope of the linear portion).

#### Photopic response

Photopic b-waves were isolated by removing the band-pass filtered OPs (−3 dB at 50 and 280 Hz). The amplitudes were then evaluated from baseline to the peak for intensities from 1.72 and 2.72 log cd.s.m^−2^.

#### Oscillatory potentials, Ops

Amplitude of scotopic and photopic OPs were measured by summing the root-mean-square (RMS) amplitude of band-pass filtered waveforms (−3 dB at 50 and 280 Hz) beginning at the trough preceding the first OP and ending at the trough following the last OP. Comparison between treated and control eyes were made for relative amplitude (treated/control, %) averaged over a range of intensities for scotopic (0.72 to 2.72 log cd.s.m^−2^) and photopic OPs (1.72 to 2.72 log cd.s.m^−2^).

#### Retinal function versus IOP

The relationship between the amplitude of each ERG component (Rm*_P3_*, P2 V*_max_*, scotopic OPs, photopic b-wave, photopic OPs, pSTR, nSTR) relative to the fellow control eye and IOP was described using an inverse cumulative normal function, as previously described [20], The function returns a measure of sensitivity to IOP elevation (μ, mmHg) as well as steepness of the transition portion (σ, mmHg) [27]. For each ERG component a non-parametric bootstrap [28] was used to establish 95% confidence interval (CI) for the µ and σ parameters of each ERG component.

#### Other statistical methods

Analysis-of-variance (ANOVA; Prism, v3.02, GraphPad Software Inc., San Diego, CA, USA) was applied to test the significance of the treatment effect, whereby the null hypothesis was no effect of IOP elevation. Two-way ANOVA (IOP “treatment” vs. ERG stimulus intensity) was applied to the data for each of the seven ERG components independently. Therefore, the alpha-level was adjusted to 0.01 to correct for multiple comparisons (i.e. to limit type-two errors given that seven ERG parameters were evaluated).

## Results

### Recovery of Rat ERG from Acute IOP Elevation

**Figure 1:**
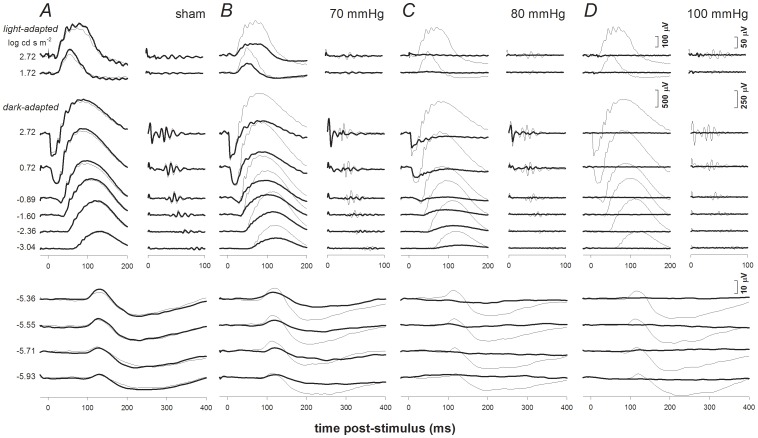
Representative individual examples of ERG findings 4 weeks after acute IOP elevation for selected groups. ERG responses for experimental eyes (bold traces) and their fellow control eyes (thin traces) are shown for the following groups: sham (Column **A**), 70 mmHg (Column **B**), 80 mmHg (Column **C**) and 100 mmHg (Column **D**). Stimulus flash intensities are listed at left for scotopic (below) and photopic (above) responses. Isolated OPs are shown to the right of corresponding waveforms.

Figure 1 provides individual examples of ERG responses from four select IOP groups 4 weeks following a single episode of acute IOP elevation. These examples illustrate results for the range of IOPs that produce distinct effects on the ERG at this 4-week recovery time point. In Figure 1, the ERG responses for a range of stimulus flash intensities (indicated at left of column A) are shown for treated (thick traces) and contralateral control eyes (thin traces). Column A shows results for an animal in the sham group, while columns B, C and D show results for animals from the 70, 80, and 100 mm Hg groups, respectively.

As previously reported [29], [30], pSTR and nSTR are discernable in all control eyes at the 4 dimmest intensities. ERG responses to flash energies from −3.04 to −1.60 log cd.s.m^−2^ are dominated by the corneal positive b-wave and show distinct oscillatory potentials (isolated on the right). Brighter stimuli (>−0.89 log cd.s.m^−2^) produce waveforms with a prominent photoreceptoral a-wave, bipolar cell b-wave and OPs. Light-adapted responses (at the top) are dominated by a corneal positive b-wave with small OPs.

Figure 1A shows that 4 weeks after sham procedure (cannulation without IOP elevation), there was little difference between sham cannulated and contralateral control ERGs. In contrast, 4 weeks after IOP elevation to 70 mm Hg (Figure 1B) all components of the ERG, with the exception of the a-wave, failed to fully recover. At 80 mmHg (Figure 1C) very little STR, b-wave and OP recovery was evident, whereas the a-wave had recovered to more than 50% of its contralateral control eye. Figure 1D shows that no ERG component recovered from IOP elevation to 100 mmHg.

### Differential Recovery of ERG Components Following Acute IOP Elevation

**Figure 2:**
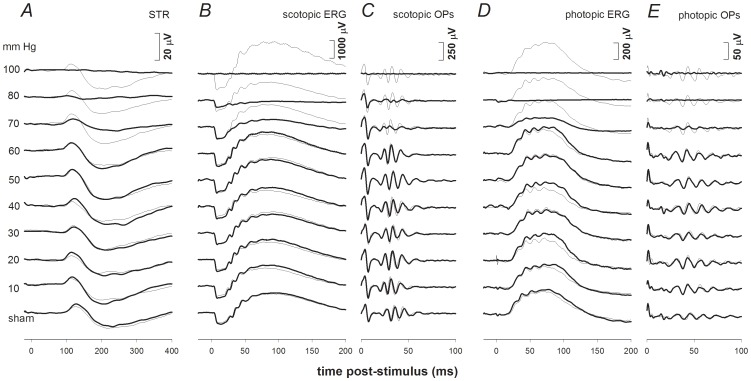
Representative ERG findings for experimental eyes (bold traces) 4 weeks after challenge for all IOP levels. Selected stimulus conditions are shown to emphasize various components of the ERG: scotopic threshold response (STR, −5.55 log cd s m^−2^, Column **A**) the scotopic bright flash ERG (2.22 log cd s m^−2^, Column **B**) and OPs (Column **C)**, as well as the photopic ERG (2.72 log cd s m^−2^, Column **D**) and Ops (Column **E**). ERG responses for fellow control eyes are shown by the thin traces in each column. IOP levels are indicated on the left.

ERG response waveforms from one representative animal in each IOP group are shown in Figure 2, which demonstrates that complete recovery of the ERG was observed for IOP levels of 60 mmHg or less. The photoreceptor a-wave showed good recovery for IOPs as high as 70 and 80 mmHg (Figure 2B), all other components only showed partial recovery for these higher levels of IOP elevation (Figure 2A–E).

**Figure 3:**
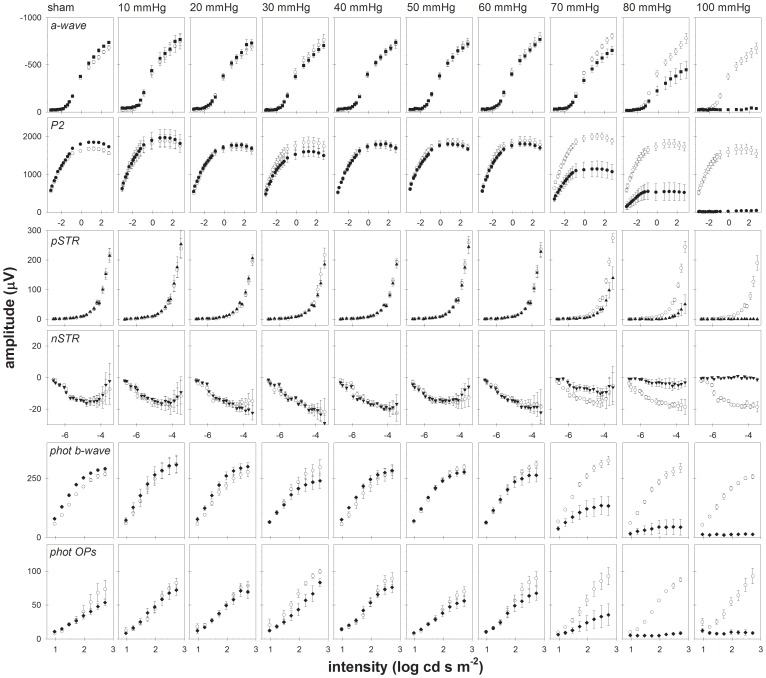
Recovery of ERG components as a function of stimulus intensity. Averaged group (± SEM) data for control eyes (unfilled symbols) and experimental eyes (filled symbols).

The complete results for all animals and all stimulus intensities are presented in Figure 3 as intensity-response series for the a-wave (top row), P2 (second row), pSTR (third row), nSTR (fourth row), photopic b-wave (fifth row) and photopic OPs (bottom row). Group mean amplitudes (± SEM) in IOP elevated eyes (filled circles) and fellow control eyes (open circles) are plotted as a function of stimulus intensity within each panel. Each column of six panels represents the results in one IOP group, beginning with the sham group at the left and progressing to higher IOP levels toward the right.

Figure 3 shows that for IOP levels of 50 mmHg and below, there was no consistent alteration of ERG amplitude 4 weeks after the acute episode of IOP elevation. However, in the group of experimental eyes elevated to 30 mmHg, the a-wave, P2, photopic b-wave and photopic OP amplitudes were all significantly smaller compared with fellow control eyes (P<0.01, ANOVA) but these effects were notably less pronounced in the groups of eyes whose IOP was elevated to 40 or 50 mmHg. This unusual pattern may represent the effect of outliers within the IOP 30 mmHg group of eyes that might have been particularly susceptible to the episode of acute IOP elevation 4 weeks earlier. At 60 mmHg the P2 and photopic OPs were significantly smaller than the fellow control eye group, a pattern that persisted and became more prominent at higher IOPs. At 70 mm Hg, all ERG components failed to fully recover (P<0.0001, ANOVA) and this effect was even more pronounced at 80 mmHg. For eyes elevated to 100 mmHg there was no recovery of any ERG component.

**Figure 4:**
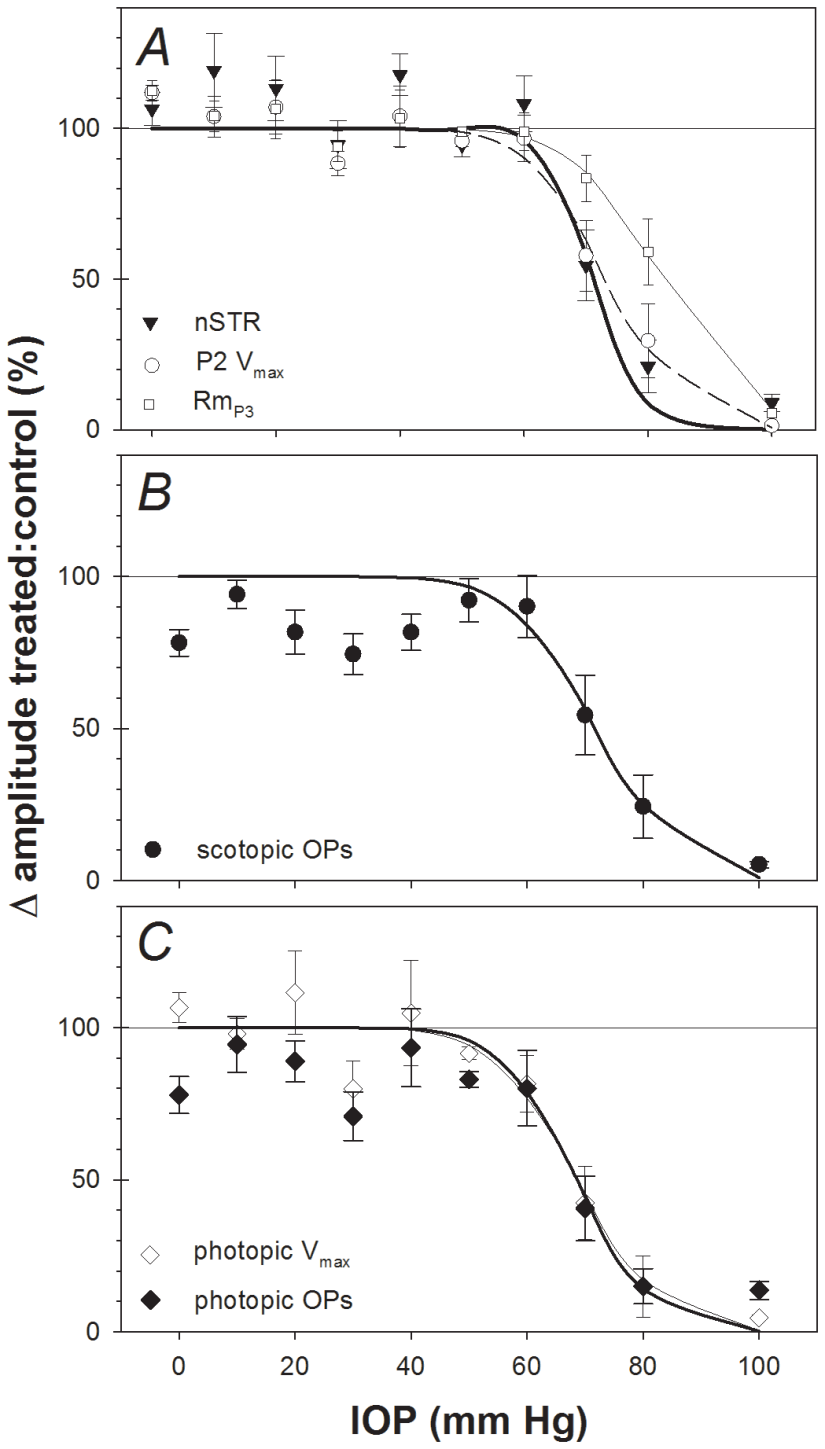
Relative change across ERG components 4 weeks following acute IOP insult. (**A**) nSTR (filled triangles), P2 amplitude (V*_max_*, open circles) and photoreceptoral amplitude (Rm*_P3_*, filled squares). For comparison, the corresponding best-fit cumulative normal functions are also plotted in panel A for the nSTR (thin solid curve), P2 (V*_max_*, dashed curve) and a-wave (Rm*_P3_*, bold curve). (**B**) Scotopic OP amplitude. (**C**) Photopic b-wave amplitude (V*_max_*, open diamonds) and photopic OP amplitude (filled diamonds). For clarity, the best-fit cumulative normal functions are omitted from panels B and C.

The relative sensitivity of each ERG component to an episode of acute IOP elevation 4 weeks earlier was evaluated by comparing parameters from the function relating relative amplitude (treated/control, %) versus IOP (see Methods and previous work) [20]. Figure 4A confirms that all major scotopic ERG components recovered 4 weeks after IOP elevation to 60 mmHg. In contrast, following IOP elevation to 70 mmHg, the a-wave recovered to 83±8% of the contralateral control eye, whereas the P2 (58±11%, pSTR (55±10%) and nSTR (55±12%) showed substantially less recovery. The difference in recovery between inner and outer retinal ERG components was even greater in the group of eyes that underwent IOP elevation to 80 mmHg. There was no recovery following IOP elevation to 100 mmHg for any ERG component. The relative response as a function of IOP for each ERG components was fit with an inverse cumulative normal function to obtain a quantitative estimate of sensitivity (position parameter, µ, and slope parameter σ) as shown in Figure 4A for the nSTR (filled triangles), scotopic P2 (open circles) and a-wave (Rm*_P3_*, open squares). Panel B shows the relative amplitude of the scotopic OPs plotted versus IOP and panel C shows the relative amplitude of the photopic b-wave (V*_max_*, open diamonds) and photopic OPs (filled diamonds) plotted versus IOP. The parameters resulting from the best fitting cumulative normal function are listed for each ERG component in Table 1.

**Table 1:** Cumulative normal parameters for relative amplitude as a function of acute IOP.

	*µ position, mmHg*			*σ slope constant*	
ERG parameter	week 4 (95% CL)	Acute (95% CL)	Change	week 4 (95% CL)	Acute (95% CL)
Rm*_P3_*	82.3* (78.5–86.5)	71.1 (68.8–73.6)	11.3	11.7 (6.6–14.3)	13.1 (9.1–17.9)
scotopic P2 V*_max_*	73.5* (70.1–78.7)	61.4 (58.5–64.2)	12.1	10.5 (6.1–15.0)	8.1 (5.2–10.7)
scotopic OPs	71.9* (66.7–76.2)	65.3 (60.7–69.6)	6.6	12.0 (5.2–32.7)	7.4 (3–10.6)
pSTR	72.3* (69.4–75.5)	58.9 (55.8–62.2)	13.3	7.5 (1.8–11.6)	11.3 (7.4–16.9)
nSTR	71.3* (69.1–73.3)	64.4 (59.5–70.5)	6.9	6.4 (1.8–8.3)	6.3 (1.5–9.1)
photopic b-wave	68.5* (65.3–72.9)	60.2 (58.4–63.3)	8.3	10.7 (5.6–17.3)	6.6 (1.5–10.3)
photopic OPs	68.7* (63.5–72.3)	55.7 (52.0–59.1)	13.0	11.9 (1.9–33.8)	16.7 (5.7–22.7)

* significant recovery p<0.05 compared with acute from non-parametric bootstrap.

Comparison across ERG parameters reveals that at this 4-week recovery time point, the photopic P2 and photopic OPs exhibit the highest sensitivity to the prior episode of acute IOP elevation. The next most sensitive ERG components were the nSTR, scotopic OPs, pSTR and scotopic P2. The scotopic a-wave (Rm*_P3_*) was the least sensitive ERG component showing 50% loss at an IOP of 82.3 mmHg. The slope parameter (σ) tended to be steeper for the nSTR and pSTR, however these slope differences were not significantly different from the other components (as the estimates overlap with the 95% confidence limits of other parameters). This pattern of observations demonstrates a greater sensitivity to acute IOP elevation of the ERG components arising from the inner retina (STR and OPs) as compared with those arising from the outer retina (photoreceptors, P3). This gradient of sensitivity observed here after 4 weeks of recovery from acute IOP elevation generally matches the results observed for ERGs recorded during the episode of acute IOP elevation [20]. Comparison of scotopic to photopic systems (e.g. for bipolar cell responses such as the V*_max_* parameter or the OPs) suggests that both systems exhibit similar sensitivity for recovery to acute IOP elevation.

**Figure 5:**
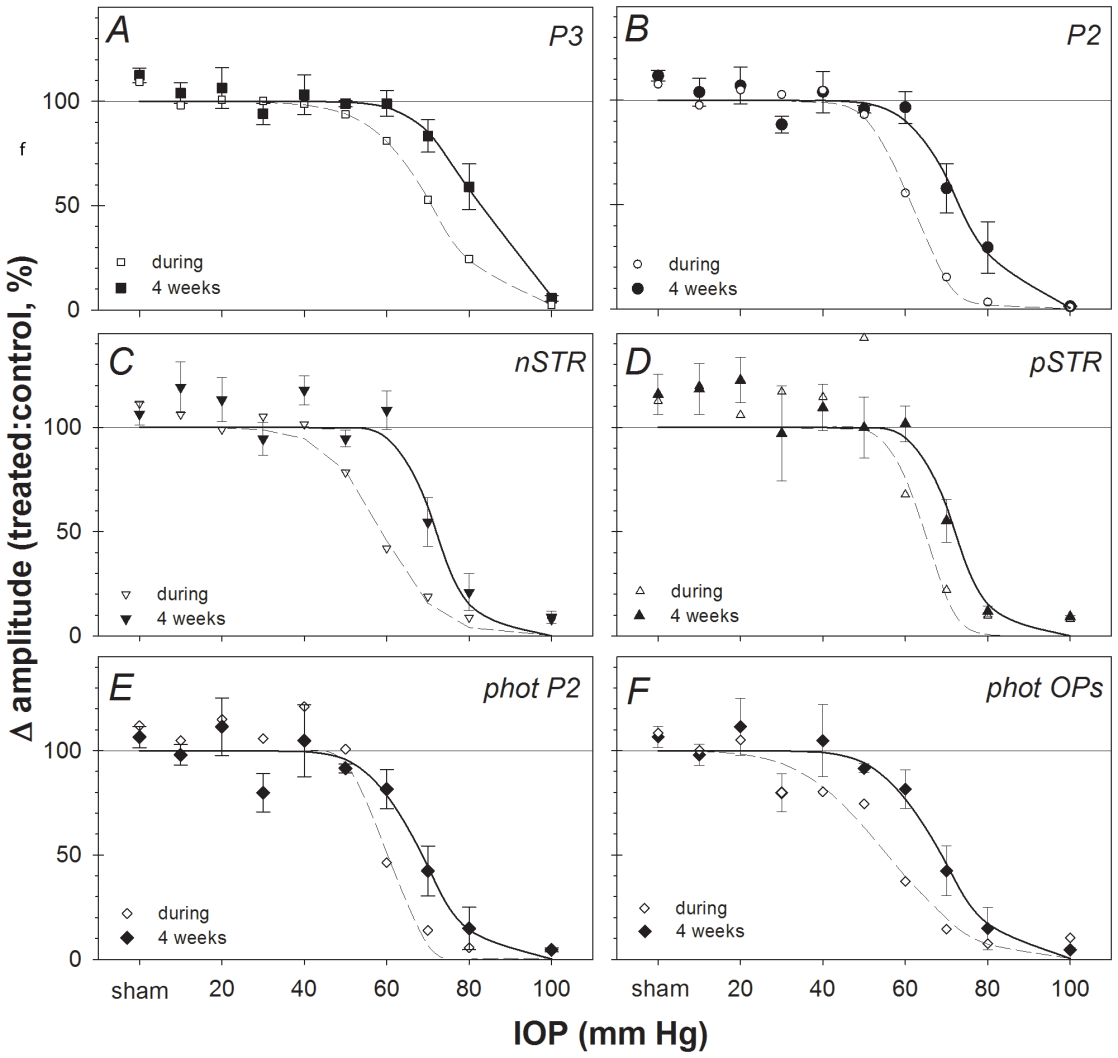
ERG component amplitudes recorded during acute IOP elevation vs. **after 4 weeks recovery.** Group mean relative amplitude (ratio of treated/fellow control eyes, %, filled symbols, ± SEM) for (**A**) a-wave (Rm*_P3_*, P3), (**B**) b-wave (V*_max_*, P2), (**C**) nSTR, (**D**) nSTR, (**E**) Photopic b-wave (V*_max_*, P2) and (**F**) photopic OP components 4 weeks following acute IOP elevation for each level of IOP (solid curves represent best-fit cumulative normal functions). For comparison, the relative amplitudes of each component recorded during acute IOP elevation are re-plotted from a previous report [20] (unfilled symbols, dashed curves represent best-fit cumulative normal functions).

For comparison, Table 1 also lists the results of the same sensitivity analysis applied to the ERGs recorded during the episode of acute IOP elevation as previously published [20]. The comparison shows that each ERG component was significantly more sensitive during the episode of acute IOP elevation than after 4 weeks of recovery, i.e. all ERG components demonstrated significant recovery as compared with the degree of dysfunction measured during the acute event. This is also shown in Figure 5, which compares component amplitudes of ERGs recorded during the period of acute IOP elevation (unfilled symbols, dashed curves) with those 4 weeks after IOP elevation (filled symbols, solid curves). The data presented in Figure 5 also clearly demonstrate that all ERG components recovered significantly 4 weeks after IOP elevation unless the latter was catastrophically high (100 mmHg). Table 1 shows that the components exhibiting the best recovery (i.e., the largest difference n μ between acute and 4 weeks) were the pSTR and photopic OPs, whereas those showing the least recovery were the scotopic OPs and the nSTR.

**Figure 6:**
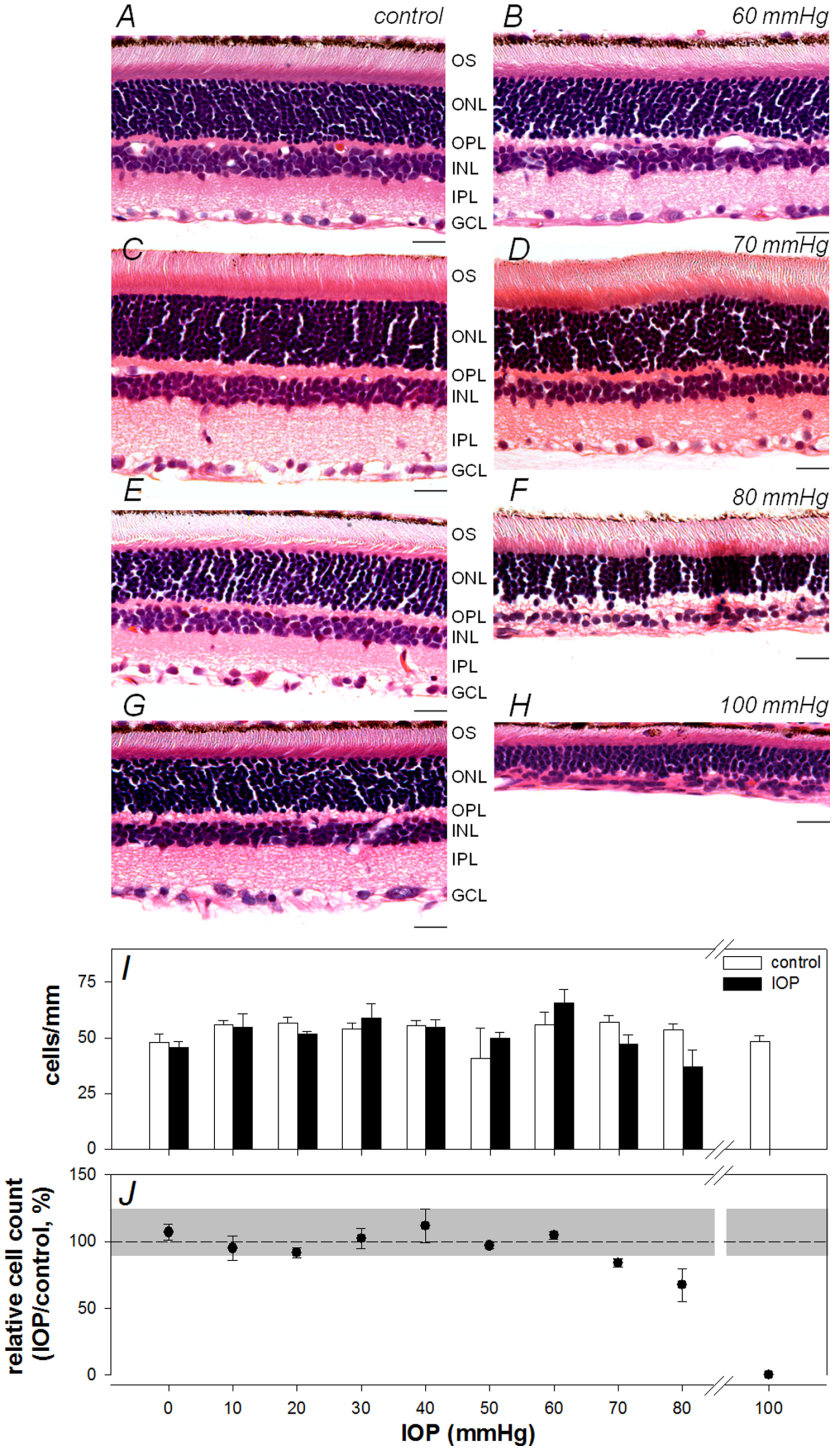
The effect of acute IOP elevation on retinal structure. Representative histological sections for selected IOP levels, 60 mmHg (**A**: control, **B**: IOP), 70****mmHg (**C**: control, **D**: IOP), 80 mmHg (**E**: control, **F**: IOP)**,** 100 mmHg (**G**: control, **H**: IOP). Scale bar = 20 µm (**I**) Mean (± SEM) RGC layer density for control (unfilled) and IOP treated eyes (filled) is shown across increasing IOP levels. (**J**) Relative RGC layer density (treated/fellow control eye, mean ± SEM, %) as a function of IOP. Grey area represents 95% confidence limits for % difference between eyes of the sham group.

Figure 6 shows representative histological sections sampled from eyes of four IOP groups: fellow control eyes are shown in panels A, C, E, G and experimental eyes are shown in panels B, D, F, H for IOP of 60, 70, 80 and 100 mmHg, respectively. Mean RGC layer density (cells/mm, ± SEM) is graphed in panel I for each IOP group along with values for the corresponding groups of fellow control eyes; normalized density (experimental eye relative to fellow control eye, %) is graphed in panel J. As the example in panel B and the summary data in panels I-J show, RGC layer density was not reduced in eyes whose IOP was 60 mmHg or less. As shown in panel J, at 70 and 80 mmHg there was a statistically significant reduction in RGC layer density (P<0.05), with a reduction of 16±3% and 33±12% at 70 and 80 mmHg, respectively. At 100 mmHg, no cells were evident within the RGC layer and the more distal retinal nuclear layers were also thinner than control eyes.

We compared the relative loss of inner retinal function (experimental:control eye) against relative loss of RGC layer density for ERG components known to arise from neurons in the RGC layer (pSTR, nSTR and OPs) [29]. For each of these ERG components, a linear model was found to be a statistically superior description of this relationship as compared with nonlinear models (such as quadratic or exponentials with offset; P>0.05; F-tests and Akaike’s Information Criterion tests). Thus we used linear regression to determine whether loss of inner retinal function was correlated with loss of neuronal density. In each case, a runs test and a D’Agostino and Pearson omnibus K2 test performed on the residuals confirmed that a linear model was appropriate. The results demonstrate that inner retinal function was significantly correlated with RGC layer density: R = 0.69, 0.74, 0.70 and 0.68 for the pSTR; nSTR, scotopic OPs and photopic OPs, respectively (P<0.0001 in each case).

## Discussion

Previously we reported that ERG components arising from the inner retina were more sensitive to acute IOP elevation than components arising from the middle and outer retina [20]. In the present study we demonstrate that this same pattern occurs 4 weeks following the episode of acute IOP elevation. However, at this 4-week recovery time point, this gradient pattern of dysfunction progressing from inner to outer retina was less prominent. In general, the photoreceptoral amplitude was least sensitive to IOP but all other ERG components showed similar sensitivity (Table 1) to each other.

A key outcome of this study was that compared with responses acquired during the acute episode of IOP elevation, all ERG components showed significant recovery 4 weeks later, as evidenced by a rightward shift of the IOP-dose response curve (Figure 5 and Table 1). This shift ranged from 6.6 to 13.3 mmHg, meaning that approximately 10 mmHg higher level of IOP was required to cause permanent functional loss for a given ERG component as compared with the abnormality measured during the acute event. Importantly, all ERG components recovered to a similar extent. It should also be noted however, that no recovery was observed subsequent to the catastrophic levels of IOP elevation to 100 mmHg (i.e. complete ischemia for 105 minutes duration) [7], [31]. The histological appearance of the retina in eyes that had IOP set to 100 mmHg was also consistent with injury arising from severe ischemia (and reperfusion). [32] Büchi et al [33] and Hughes et al [34] assessed retinal histology in rat retina following IOP elevation to levels near or above systemic blood pressure (110 and 155 mmHg, respectively) and showed that the degree of retinal thinning was associated with duration of injury. Our ganglion cell loss at the highest IOP level is consistent with reports by these authors in different strains of rats.

For IOPs set to 60 mmHg or below (for 105 minutes) all aspects of retinal function (ERG components) showed complete recovery. This suggests that an IOP of 70 mmHg (or an ocular perfusion pressure [OPP = BP–IOP] of ∼28±11 mmHg or lower) for 105 minutes is sufficient to produce permanent retinal dysfunction in the rat retina. The extent of permanent retinal functional loss was progressively worse for higher levels of IOP. Significant reduction in retinal blood flow has been found at an IOP of 70 mmHg in rats [6], [7], [31]. Both He et al [7] and Zhi et al [31] report that 70 mmHg produces about a 70% reduction of retinal blood flow. It is interesting to speculate, given our functional outcomes and the retinal blood flow data of He et al [7] and Zhi et al [31] that reduction of retinal blood flow that does not exceed ∼50% of normal does not lead to permanent retinal dysfunction (at least if it does not persist for more than a few hours).

There was a strong association between permanent loss of inner retinal functional and loss of cells in the RGC layer and a similar IOP ‘threshold’ between 60 and 70 mmHg above which both permanent functional loss of cells from the RGC layer were observed. This adds further evidence to support a common mechanism for the origin of damage – a reduction in local blood flow during acute IOP injury (as discussed in the preceding paragraph). For IOP of 70 mmHg or more, Holcombe et al [35] found that histological damage was associated with inner retinal hypoxia as indicated by the presence of hypoxyprobe-labelled cells in proximal rat retina. Furthermore, Selbach et al [36] reported that IOP of 70 or greater produce reductions in vessel diameter and oxygen saturation in rabbit optic nerve. Such reduction in blood supply and oxygen saturation are consistent with impaired oxidative metabolism at or above 70 mmHg, as indicated by a reduction in vitreal pH at this pressure in the rabbit model of acute IOP elevation [37]. Taken together, our current results and previously published evidence suggest that the IOP threshold for permanent retinal dysfunction is associated with vascular and/or metabolic insufficiency. At severe levels of ischemia - above systemic blood pressure (>110 mmHg) - the greater susceptibility of the inner retina compared with the outer retina may be species dependent. Greater cell loss has been observed in the outer than the inner retina after ischemia-reperfusion in primate [38] and rabbit [39] retinas. Uckerman and colleagues [39] reported that outer retinal sensitivity is associated with exudative retinal detachment following ischemia-reperfusion. The presence of retinal detachment may account for the different outcomes between our study and those in rabbit eyes.

Previous studies have shown that the capacity and time required for particular ERG components to recover following acute IOP elevation [40], [41] depends on the magnitude and duration of IOP elevation [40]. He et al [40] showed that after a spike of 70 mmHg (OPP, 33±14 mmHg) for either 15 or 30 minutes, inner retinal function (monitored by the nSTR) fully recovered within 60 and 90 minutes, respectively. In contrast, for a longer insult of 60-minutes, the nSTR amplitude had only recovered to 80% at 120 minutes after the end of the IOP spike. The results of the current study are consistent with these other previous studies [40], [42] in that there appears to be an interaction between the magnitude and duration of IOP elevation, which collectively define a threshold for permanent damage. Hughes et al [34] showed that for an IOP of 155 mmHg, 30 minutes of insult was not enough to produce retinal thinning. There is a very narrow window spanning these dimensions within which damage is selective for RGCs (such as occurs for the vast majority of human glaucoma cases). Unfortunately it is not uncommon for IOP in rodent experimental glaucoma models to reach levels beyond the threshold for non-selective retinal damage, but the results presented and discussed here suggest that large IOP spikes (i.e. above 60 mmHg) should be avoided. Whilst the rodent model has become commonly used for studies of ischemia and IOP elevation, there are potentially important differences between the vasculature and optic nerve of this species and humans [43], [44]. Whether the same damage mechanisms and threshold levels for acute episodes of IOP elevation exist in human eyes requires further investigation.

### Conclusions

We found that for IOP lower than 100 mmHg there is some degree of retinal functional recovery compared with the attenuation observed during the acute episode of IOP elevation. All ERG components showed a similar degree of recovery. Importantly the results also show that there is a ‘threshold’ for permanent retinal damage in the rat at an IOP of 60–70 mmHg if sustained for 105 minutes or more.
